# Treatment of Post-Neurosurgical Ventriculitis and Scalp Infection Caused by Klebsiella pneumoniae Carbapenemase and New Delhi Metallo-β-Lactamase Co-Producing Carbapenem-Resistant Klebsiella pneumoniae: A Case Report

**DOI:** 10.7759/cureus.109007

**Published:** 2026-05-17

**Authors:** Xin ru Ma, Song yu Chen, Run hai Wu, Qiao Li, Jin ming Zhang, Min Ni, Fuming Shen

**Affiliations:** 1 Department of Pharmacy, Nanjing Medical University, Nanjing, CHN; 2 Department of Neurosurgery, Shanghai Tenth People's Hospital, Shanghai, CHN; 3 Department of Pharmacy, Shanghai Tenth People's Hospital, Shanghai, CHN; 4 School of Pharmacy, Nanjing Medical University, Nanjing, CHN; 5 Department of Pharmacy, Shanghai Tenth People's Hospital, Tongji University School of Medicine, Shanghai, CHN

**Keywords:** aztreonam, central nervous system infection, meropenem-vaborbactam, pharmacokinetics, therapeutic drug monitoring

## Abstract

The treatment of intracranial infections caused by* Klebsiella pneumoniae* carbapenemase and New Delhi metallo-β-lactamase coproducing carbapenem-resistant *Klebsiella pneumoniae* is extremely limited. The blood-brain barrier severely restricts the penetration of most systemically administered antibiotics, and robust pharmacokinetic/pharmacodynamic data for novel agents in the central nervous system remain scarce. Therefore, identifying effective therapeutic strategies is critical. We report a study of a 36-year-old Han Chinese male treated with meropenem-vaborbactam and aztreonam in the case of post-neurosurgical ventriculitis and scalp infection caused by *Klebsiella pneumoniae* carbapenemase and New Delhi metallo-β-lactamase co-producing carbapenem-resistant *Klebsiella pneumoniae*. Meanwhile, we also examined the plasma and cerebrospinal fluid concentrations of meropenem-vaborbactam at 0, 1, 2, 3, 5, and 8 h over a complete dosing interval. Pharmacokinetic analysis was as follows: for meropenem and vaborbactam, the plasma concentrations were 0.75-36.33 mg/L and 4.57-94.71 mg/L, respectively, while those in cerebrospinal fluid ranged from 1.04 to 4.66 mg/L and 3.29 to 16.11 mg/L, respectively. Calculated cerebrospinal fluid penetration reached 19.0% for meropenem and 21.2% for vaborbactam. This work further emphasizes the significance of therapeutic drug monitoring in clinical practice in order to optimize antibiotic therapy in severe infections.

## Introduction

Intracranial infection is one of the most severe complications following neurosurgery [1]. According to the 2024 report from the China Antimicrobial Drug Surveillance Network (CHINET), among 3,360 cerebrospinal fluid (CSF)-positive samples collected in China, *Acinetobacter baumannii* accounted for 12.3% and *Klebsiella pneumoniae* for 11.0%. The increasing incidence of intracranial infections caused by multidrug-resistant (MDR) and extensively drug-resistant (XDR) Gram-negative bacteria has become a major clinical challenge, largely driven by the widespread use of broad-spectrum antibiotics [2].

Meropenem-vaborbactam (MVB) is a novel carbapenem/β-lactamase inhibitor combination with potent activity against serine carbapenemases, particularly *Klebsiella pneumoniae carbapenemase* (KPC)-producing strains. However, MVB has limited activity against metallo-β-lactamases such as New Delhi metallo-β-lactamase (NDM) [3]. The recent emergence of KPC and NDM co-producing carbapenem-resistant *Klebsiella pneumoniae* (KPC-NDM-CRKP) has further complicated antimicrobial treatment, because these strains are resistant to many currently available β-lactam antibiotics [4]. In vitro studies have shown that combining aztreonam (ATM) with β-lactamase inhibitors such as avibactam or vaborbactam may restore antimicrobial activity against NDM-producing organisms [5]. Nevertheless, clinical evidence supporting this strategy, particularly for central nervous system (CNS) infections, remains extremely limited.

Here, we report a rare case of post-neurosurgical ventriculitis and scalp infection caused by KPC-NDM-CRKP that was successfully treated with a combination of MVB and ATM. In addition, we characterized plasma and CSF MVB concentrations at multiple time points over the complete dosing interval and analyzed their pharmacokinetic parameters. To the best of our knowledge, this is the first report to describe both the clinical efficacy and CSF pharmacokinetics of MVB in a patient with KPC-NDM-CRKP CNS infection. This case may therefore provide clinically relevant evidence for the management of highly resistant Gram-negative CNS infections.

## Case presentation

A 36-year-old Han Chinese male presented to a local hospital after a fall at home with altered consciousness and unresponsiveness. The patient had no past medical history, surgical history, pertinent family history, or regular medication use. On admission, he exhibited bilateral pupillary dilation and underwent emergency endotracheal intubation. Cranial imaging revealed a left frontal intracranial hematoma, and emergency hematoma evacuation with decompressive craniectomy was performed.

Postoperatively, the patient was admitted to the intensive care unit (ICU) for intracranial pressure management and infection prevention. However, his neurological recovery remained poor, and he developed a persistent high fever (maximum temperature 39.5°C) several days after surgery. Concurrently, purulent discharge from the scalp incision and progressive deterioration in consciousness were observed.

Subsequently, both CSF and sputum cultures yielded CRKP. CSF analysis demonstrated marked inflammatory changes characterized by neutrophilic pleocytosis, elevated protein levels, and decreased glucose concentration. Given the patient’s postoperative neurosurgical status and the presence of ventricular drainage devices, these findings strongly raised suspicion for postoperative intracranial infection rather than isolated pulmonary infection or postoperative inflammatory response. Differential diagnoses initially included postoperative aseptic meningitis, ventilator-associated pneumonia, surgical site infection, and central fever after traumatic brain injury. Targeted antimicrobial therapy with colistin was therefore initiated.

The patient was later transferred to our hospital for further management. On admission, his body temperature was 39.8°C, and his blood pressure was 134/76 mmHg, while other vital signs were within normal limits. Physical examination revealed a coma with a Glasgow Coma Scale score of 3. Laboratory testing showed leukocytosis (white blood cell count 20.45 × 10⁹/L) with neutrophil predominance (87.7%), whereas renal and hepatic function remained normal. Given the persistent fever, purulent wound secretion, CSF abnormalities, and previous CRKP isolation, postoperative ventriculitis with wound involvement was strongly suspected.

Emergency neurosurgical intervention under general anesthesia was performed, including (1) debridement of the craniocerebral wound, (2) placement of an external ventricular drain (EVD) with an Ommaya reservoir, and (3) repair of cerebrospinal fluid leakage. On day seven, based on antimicrobial susceptibility testing of CRKP isolated from CSF (amikacin MIC ≤ 2 mg/L, colistin MIC ≤ 0.5 mg/L, ceftazidime-avibactam (CAZ-AVI) disk diffusion zone diameter 26 mm), treatment with CAZ-AVI (2.5 g q8h IV), amikacin (0.6 g qd IV), and intraventricular colistin (100,000 IU qd) was initiated.

**Figure 1:**
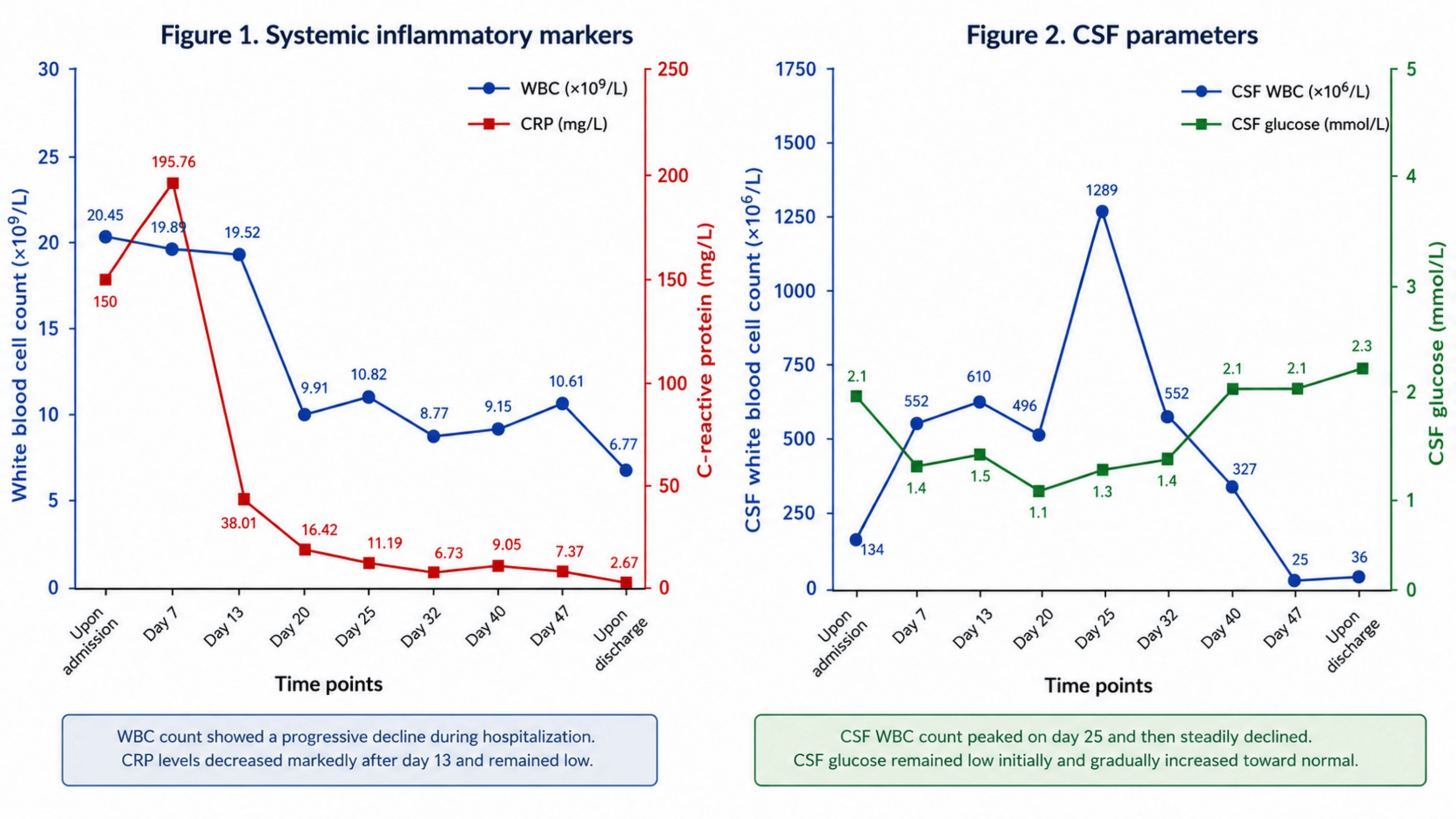
Serial changes in systemic inflammatory markers and cerebrospinal fluid (CSF) parameters during hospitalization. (Left panel) Systemic inflammatory markers: white blood cell (WBC) count (blue line, ×10⁹/L) and C-reactive protein (CRP) level (red line, mg/L) gradually decreased during antimicrobial treatment. (Right panel) CSF parameters: CSF white blood cell count (blue line, ×10⁶/L) peaked on day 25 and subsequently declined, whereas CSF glucose (green line, mmol/L) gradually returned toward normal levels. CRP = C-reactive protein. CSF = cerebrospinal fluid. WBC = white blood cell.

**Figure 2:**
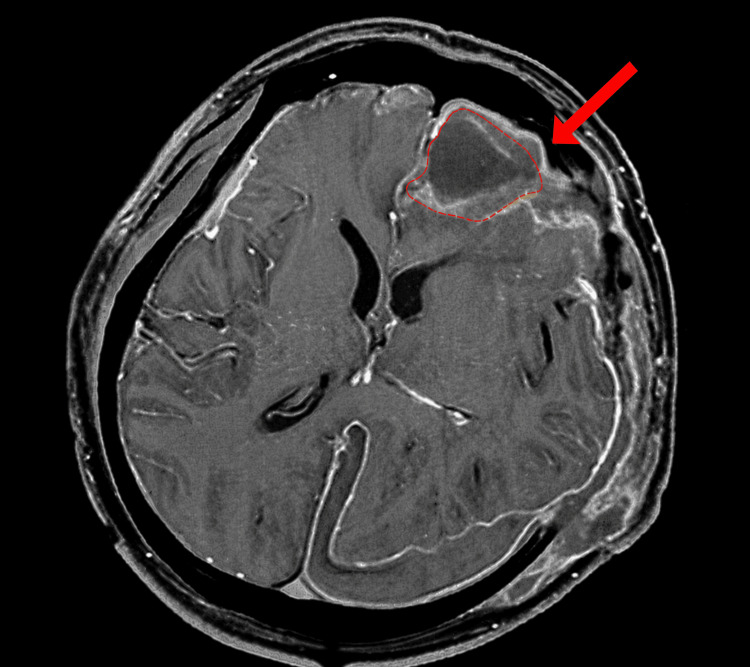
Axial post-contrast T1-weighted MRI of the brain. The red arrow and dashed outline highlight the heterogeneously contrast-enhancing lesion in the left frontal lobe.

Six days later, KPC-NDM-producing CRKP was identified in the CSF. The isolate remained susceptible to colistin (MIC ≤ 0.5 mg/L), tigecycline (MIC = 2 mg/L), and amikacin (MIC ≤ 2 mg/L) but was resistant to CAZ-AVI (disk diffusion zone diameter 19 mm). Antimicrobial susceptibility profiles of *Klebsiella pneumoniae* isolates are summarized in Table 1. Repeat CSF examination demonstrated severe inflammatory abnormalities, including a white blood cell count of 610 × 10⁶/L, protein concentration of 2833 mg/L, glucose level of 1.5 mmol/L, and neutrophil proportion of 83%. Changes during the course of treatment are shown in Figure 1. MRI further revealed extensive subcutaneous and intracranial postoperative infection, predominantly involving the left surgical region (Figure 2). Based on the microbiological, laboratory, and imaging findings, the patient was diagnosed with post-neurosurgical ventriculitis and scalp infection caused by KPC-NDM-CRKP.

**Table 1:** Antibiotic susceptibilities of Klebsiella pneumoniae in the patient's CSF cultures. MIC = minimum inhibitory concentration. K–B = Disk diffusion test (K–B method). CSF = cerebrospinal fluid.

Antibiotic	MIC (mg/liter) or K–B (mm)	Interpretation
Piperacillin/tazobactam	＞=128	Resistant
Ceftazidime	＞=64	Resistant
Cefoperazone-Sulbactam	＞=64	Resistant
Cefepime	＞=32	Resistant
Aztreonam	＞=64	Resistant
Imipenem	＞=16	Resistant
Meropenem	8	Resistant
Amikacin	＜=2	Susceptible
Tobramycin	＞=16	Resistant
Ciprofloxacin	＞=4	Resistant
Levofloxacin	＞=8	Resistant
Doxycycline	4	Susceptible
Minomycin	4	Susceptible
Tigecycline	2	Susceptible
Colistin	＜=0.5	Susceptible
Compound Sulfamethoxazole	＜=20	Susceptible
Ampicillin	6	Resistant
Ampicillin-Sulbactam	6	Resistant
Cefazolin	6	Resistant
Cefuroxime	6	Resistant
Cefoxitin	6	Resistant
Ceftriaxone	6	Resistant
ESBL	+	+
Fosfomycin	6	Resistant
Ceftazidime-avibactam	19	Resistant
KPC	+	+
NDM	+	+

**Figure 3:**
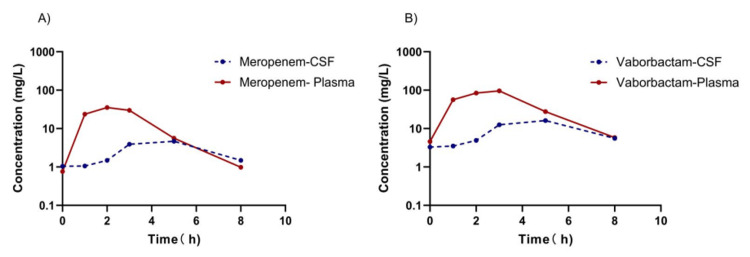
Concentration of meropenem/vaborbactam in the patient’s plasma and CSF on day three after the first intravenous infusion. Figure 3. Concentration–time profiles of meropenem/vaborbactam in plasma and CSF on day three after the first intravenous infusion. (A) Meropenem concentrations in plasma (red solid line) and CSF (blue dashed line) over a 0–8 h dosing interval. (B) Vaborbactam concentrations in plasma (red solid line) and CSF (blue dashed line) over a 0–8 h dosing interval. Drug concentrations are expressed in mg/L, and time is presented in hours (h). The y-axis is displayed on a logarithmic scale (0.1–1000 mg/L) to better illustrate differences between plasma and CSF exposure. Both meropenem and vaborbactam demonstrated measurable penetration into the CSF throughout the dosing interval, with delayed peak concentrations and lower overall exposure in CSF compared with plasma, consistent with limited but sustained central nervous system distribution. CSF = cerebrospinal fluid.

**Figure 4:**
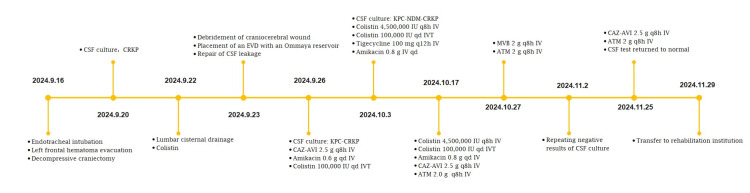
Timeline from symptom onset to recovery.

The antibiotic regimen was subsequently modified to colistin (4,500,000 IU q8h IV and 100,000 IU qd IVT), tigecycline (100 mg q12h IV), and amikacin (0.8 g qd IV). On day 25, KPC-NDM-CRKP was still cultured from the patient's CSF, and the CSF biochemical parameters did not improve. The antibiotic regimen was adjusted to include colistin (4,500,000 IU q8h IV and 100,000 IU qd IVT), amikacin (0.8 g qd IV), CAZ-AVI (2.5 g q8h IV), and ATM (2.0 g q8h IV). On day 35, the patient's temperature increased to 38°C. Meanwhile, CSF analysis revealed elevated protein concentration (2570 mg/L), elevated white blood cell count (1289 × 10⁶/L), and reduced glucose concentration (1.3 mmol/L). We attributed the treatment failure to the inadequate activity of CAZ-AVI against KPC-producing strains. Therefore, the antibiotic regimen was changed to MVB (2 g q8h IV) and ATM (2 g q8h IV) to address the infection. The plasma and CSF concentrations of meropenem and vaborbactam were measured using a validated liquid chromatography-tandem mass spectrometry method, and pharmacokinetic/pharmacodynamic (PK/PD) analysis was conducted to evaluate MVB in plasma and CSF collected on the third day of MVB administration (Figure 3). Negative CSF cultures on post-treatment days five and six demonstrated microbiological clearance. After 30 days of combination therapy with MVB and ATM, CSF analysis revealed normalization of protein concentration, white blood cell count, and glucose concentration. Subsequently, CAZ-AVI (2.5 g q8h IV) and ATM (2 g q8h IV) were administered for sequential treatment. On day 67, the patient was transferred from the ICU (Figure 4).

## Discussion

Bacterial infections caused by CRKP are one of the major threats to global public health [6]. Few antibiotics remain effective against CRKP. Avibactam is effective against KPC but does not inhibit NDM [7]. However, the emergence of KPC-NDM-CRKP exacerbates the challenges faced in clinical practice [8]. Treating infections caused by KPC-NDM-CRKP is particularly challenging due to the synergistic enzymatic resistance resulting from the coexistence of two distinct carbapenemase classes. KPC, a class A serine β-lactamase, hydrolyzes nearly all β-lactams, including penicillins, cephalosporins, carbapenems, and monobactams. NDM, a class B metallo-β-lactamase, inactivates all β-lactams except for monobactams. When both enzymes are co-produced, their complementary hydrolytic profiles result in pan-β-lactam resistance, leaving virtually no β-lactam-based therapeutic options [9]. This dual resistance mechanism severely limits the efficacy of last-resort antibiotics, including carbapenems and novel β-lactam/β-lactamase inhibitor combinations [10]. In this case, we describe a patient with simultaneous ventriculitis and scalp infection caused by KPC-NDM-CRKP. Based on a few successful case reports [11-13], we administered colistin (4,500,000 IU q8h IV and 100,000 IU qd IVT), amikacin (0.8 g qd IV), CAZ-AVI (2.5 g q8h IV), and ATM (2.0 g q8h IV) for the initial treatment. However, after 10 days of this regimen, the patient showed no clinical improvement, with persistent fever and abnormal CSF parameters.

Vaborbactam is more potent against KPC than avibactam, which requires higher doses to achieve comparable efficacy [14]. A prospective clinical trial demonstrated that MVB exhibits more potent in vitro activity compared to other antibiotics, including meropenem, CAZ-AVI, and tigecycline [15]. Moreover, MVB retains activity against CAZ-AVI-resistant strains and has been used to treat infections caused by KPC variants [16]. In our case, the patient's CSF cultures identified KPC-NDM-CRKP with CAZ-AVI resistance. A PubMed search identified a few cases of adult patients with intracranial infections caused by CRKP successfully treated with MVB. Choi et al. [17] and Rubino et al. [18] used MVB monotherapy (2 g q8h as a 3-hour infusion). Rezzonico et al. [19] combined MVB with intraventricular gentamicin. Giuliano et al. [20] reported two cases of shunt-related ventriculitis caused by CRKP, both treated with intravenous MVB (2 g q8h via extended or continuous infusion) combined with fosfomycin in one case and ciprofloxacin in the other. Given the failure of prior therapy, we administered intravenous MVB in combination with ATM. After 30 days of combination therapy, the KPC-NDM-CRKP strain was successfully eradicated, and the patient showed significant improvement in both clinical symptoms and CSF parameters.

For the treatment of ventriculitis, antibiotic efficacy is evaluated based on CSF penetration [21]. Vaborbactam has a molecular weight of 297.14 g/mol and exhibits 33% protein binding [22], suggesting potential CSF exposure. Volpicelli et al. demonstrated that the CSF concentration of vaborbactam could reach 5-10 μg/ml after 2 days of administration of vaborbactam (2 g q8h) [16]. In this study, we conducted the first measurement of MVB concentrations in plasma and CSF at 0, 1, 2, 3, 5, and 8 h across a complete dosing interval. The pharmacokinetic parameters demonstrated that vaborbactam adequately penetrates the CSF. In our case, the CSF penetration was 21.2% (AUC_ 0-8, CSF_ / AUC _0-8, plasma_). Preclinical in vitro studies have demonstrated that a fixed vaborbactam concentration of 8 mg/L can substantially reduce the meropenem MIC_90 _against KPC-producing Enterobacterales from >32 mg/L to 1 mg/L [23]. Subsequently, this 8 mg/L concentration was adopted by the European Committee on Antimicrobial Susceptibility Testing as a reference concentration during the establishment of MVB susceptibility breakpoints [24]. In the present case, the C_max _of vaborbactam reached 16.11 mg/L, exceeding the 8 mg/L reference concentration commonly used in previous preclinical PK/PD studies and MVB breakpoint analyses. These findings indicate that vaborbactam can achieve sustained CNS exposure under inflammatory conditions and may partly explain the favorable microbiological and clinical response observed following initiation of MVB plus ATM therapy. The pharmacokinetic parameters of vaborbactam are presented in Table 2.

**Table 2:** Pharmacokinetic parameters of Farobarbatan. Cmax= Maximum concentration. Cmin= Minimum concentration. Tmax= Time to reach maximum concentration. T1/2= Elimination half-life. AUC0-8= Area under the concentration-time curve from 0 to 8 hours. CSF= Cerebrospinal fluid.

	Plasma parameter	CSF parameter
C_max_(mg/L)	94.71	16.11
C_min _(mg/L)	4.57	3.29
T_max_ (h)	4	5
T_1/2_(h)	1.34	1.96
AUC_0-8_	365.59	77.53

Meropenem exhibits high inter-individual pharmacokinetic (PK) variability, with a median CSF penetration ratio of 9% [25]. In our case, CSF penetration was 19.0% (AUC 0-8, CSF / AUC 0-8, plasma), consistent with reports in both adult ventriculitis cohorts and pediatric populations [26,27]. This suggests that, for ventriculitis, it may be necessary to increase the meropenem dose to achieve higher concentrations in the CSF [28]. The pharmacokinetic parameters of meropenem are presented in Table 3.

**Table 3:** Pharmacokinetic parameters of Meropenem. Cmax= Maximum concentration. Cmin= Minimum concentration. Tmax= Time to reach maximum concentration. T1/2= Elimination half-life. AUC0-8= Area under the concentration-time curve from 0 to 8 hours. CSF= Cerebrospinal fluid.

	Plasma parameter	CSF parameter
C_max_(mg/L)	35.33	4.66
C_min _(mg/L)	0.76	1.04
T_max_ (h)	3	5
T_1/2_(h)	1.19	1.81
AUC_0-8_	119.7	22.81

Compared to monotherapy, combination therapy offers greater benefits for CRKP infections, particularly in cases of multisite or severe infections [29]. Tiseo et al. reported two cases of infections caused by NDM-producing *Klebsiella pneumoniae* that were treated with MVB plus ATM, and assessed their synergy using the checkerboard assay [30]. Although no combined drug susceptibility testing was performed in our case, the microbiological eradication of KPC-NDM-CRKP and the improvement in CSF parameters support the efficacy of this regimen. We speculated that NDM activity was inhibited by ATM, while KPC activity was inhibited by vaborbactam. Additionally, vaborbactam may have reduced the MIC of meropenem to the extent that even minimal penetration could achieve effective clinical therapeutic outcomes.

This study also has several limitations. First, the combined drug susceptibility of MVB and ATM was not tested, preventing an assessment of the therapy's efficacy. Second, the plasma and CSF concentrations of ATM were not tested. Finally, genetic sequencing was not performed on the KPC-NDM-CRKP strain.

## Conclusions

This case provides preliminary, hypothesis-generating insights into the treatment of CNS infections. To our knowledge, it is the first report of successful treatment of post-neurosurgical ventriculitis and scalp infection caused by KPC-NDM-CRKP and the first to measure MVB concentrations in both plasma and CSF over a complete dosing interval. Our findings suggest that MVB can achieve clinically relevant CSF exposure and may contribute to favorable clinical and microbiological outcomes in patients with complicated CNS infections when combined with ATM. However, these observations are derived from a single case and should be interpreted as hypothesis-generating. Further clinical studies and PK/PD investigations with larger sample sizes are required to validate the role of MVB in CNS infections.
