# Correction: Treatment of Post-Neurosurgical Ventriculitis and Scalp Infection Caused by Klebsiella pneumoniae Carbapenemase and New Delhi Metallo-β-Lactamase Co-Producing Carbapenem-Resistant Klebsiella pneumoniae: A Case Report

**DOI:** 10.7759/cureus.c448

**Published:** 2026-06-09

**Authors:** Xin ru Ma, Song yu Chen, Run hai Wu, Qiao Li, Jin ming Zhang, Min Ni, Fuming Shen

**Affiliations:** 1 Department of Pharmacy, Nanjing Medical University, Nanjing, CHN; 2 Department of Neurosurgery, Shanghai Tenth People's Hospital, Shanghai, CHN; 3 Department of Pharmacy, Shanghai Tenth People's Hospital, Shanghai, CHN; 4 School of Pharmacy, Nanjing Medical University, Nanjing, CHN; 5 Department of Pharmacy, Shanghai Tenth People's Hospital, Tongji University School of Medicine, Shanghai, CHN

This article has been corrected to update Dr. Fuming Shen’s affiliations to: School of Pharmacy, Nanjing Medical University, Nanjing, China and Department of Pharmacy, Shanghai Tenth People's Hospital, Tongji University School of Medicine, Shanghai, China. Dr. Fuming Shen has also been made the corresponding author.

